# Unraveling the role of histone acetylation in sepsis biomarker discovery

**DOI:** 10.3389/fmolb.2025.1582181

**Published:** 2025-04-30

**Authors:** Feng Cheng, Juxin Deng, Zhaoyang Du, Lei Li, Zhaolei Qiu, Min Zhu, Hongchang Zhao, Zhenjie Wang

**Affiliations:** ^1^ Department of Emergency Surgery, The First Affiliated Hospital of Bengbu Medical University, Bengbu, Anhui, China; ^2^ School of Life Science, Anhui Agriculture University, Hefei, China; ^3^ Institute of Emergency and Critical Care Medicine, The First Affiliated Hospital of Bengbu Medical University, Bengbu, China

**Keywords:** biomarkers, histone acetylation, Mendelian randomization, sepsis, single-cell RNA sequencing

## Abstract

**Introduction:**

Sepsis is a life-threatening condition caused by a dysregulated immune response to infection. Despite advances in clinical care, effective biomarkers for early diagnosis and prognosis remain lacking. Emerging evidence suggests that histone acetylation plays a crucial role in the pathophysiology of sepsis.

**Methods:**

Transcriptomic and single-cell RNA sequencing data were used to identify histone acetylation-related genes. Differential expression analysis and weighted gene co-expression network analysis (WGCNA) were performed, followed by machine learning algorithms (LASSO, SVM-RFE, and Boruta) to screen for potential biomarkers. Mendelian randomization (MR), RT-qPCR, and functional assays were conducted for validation.

**Results:**

*BLOC1S1*, *NDUFA1*, and *SFT2D1* were identified as key biomarkers. A predictive nomogram demonstrated strong diagnostic potential. Immune infiltration and single-cell analyses linked the biomarkers to macrophage activity. MR analysis confirmed *SFT2D1* as a causal factor in sepsis. Functional assays showed that knockdown of *SFT2D1* suppressed *CXCL10* and *IL-6* expression, indicating its pro-inflammatory role.

**Discussion:**

This study identifies novel biomarkers associated with histone acetylation and immune dysregulation in sepsis. These findings deepen our understanding of sepsis pathogenesis and may facilitate the development of improved diagnostic and therapeutic strategies.

## 1 Introduction

Sepsis is a severe, life-threatening systemic inflammatory condition that arises from a dysregulated host response to infection. It is a major global health issue, with high incidence and mortality rates, particularly in critically ill patients (Singer et al., 2016). Sepsis can lead to multi-organ dysfunction, with the lungs being one of the most vulnerable organs, often affected early in the disease process (Su et al., 2024). Despite advances in antimicrobial therapy and organ support, sepsis remains the leading cause of death in intensive care units. Early diagnosis and intervention are crucial to improving patient outcomes, yet effective biomarkers for the early detection and prognosis of sepsis are still lacking.

Recent research has highlighted the critical role of epigenetic modifications, particularly histone acetylation, in the progression of sepsis. Histone acetylation, a reversible post-translational modification catalyzed by histone acetyltransferases and deacetylases, influences chromatin structure and gene expression (Wu et al., 2024). This process has profound effects on immune cell function and contributes to immune reprogramming during sepsis. Dysregulation of histone acetylation can alter the host’s immune responses, leading to persistent immune suppression and chronic inflammation (Li et al., 2024a; Li et al., 2024b; Sun et al., 2021). Understanding the mechanisms of histone acetylation in sepsis could provide new avenues for developing diagnostic biomarkers and therapeutic targets.

In this study, we utilized bioinformatics and single-cell RNA sequencing (scRNA-seq) to identify histone acetylation-related biomarkers in sepsis and elucidate their molecular and immunological mechanisms. By integrating transcriptomic data with Mendelian randomization (MR) analysis and immune cell profiling, we aimed to uncover critical pathways and regulatory networks associated with these biomarkers. These findings could contribute to improving early diagnosis, risk stratification, and personalized therapeutic approaches for sepsis.

## 2 Materials and methods

### 2.1 Data collection

The training cohort (GSE95233), validation cohort (GSE65682), and single-cell RNA sequencing (scRNA-seq) dataset (GSE167363) for sepsis were downloaded from the Gene Expression Omnibus (GEO) database (https://www.ncbi.nlm.nih.gov/geo). Specifically, the GSE95233 dataset, was utilized the GPL570 platform, included blood samples from 22 controls and 102 sepsis (Tabone et al., 2018; Venet et al., 2017). The header of the GSE95233 dataset was described as the robust multi-array average (RMA) signal intensity (log base 2). The GSE65682 dataset, was based on the GPL13667 platform, comprised blood samples from 42 controls and 51 sepsis (Scicluna et al., 2015; van Vught et al., 2016). The header of the GSE65682 dataset was described as the RMA normalized log2 transformed values. The scRNA-seq dataset GSE167363, was derived the GPL24676 platform, contained peripheral blood mononuclear cells from 2 controls and 10 sepsis (Qiu et al., 2021). Detailed information about the subjects in the three datasets were shown in Supplementary Tables S1–S3. Additionally, 77 histone acetylation-related genes (HARGs) were obtained from the literature (Qin et al., 2024).

### 2.2 Differential expression analysis

The limma package (v 3.52.4) (Ritchie et al., 2015) was utilized to perform differential analysis between sepsis and control samples on the training cohort. The threshold for differentially expressed genes (DEGs) was set |log_2_ fold change| > 0.5 and *P* < 0.05. The ggplot2 package (v 3.3.6) (Xu et al., 2024) was used to plot the volcano plot, and the ComplexHeatmap package (v 2.20.0) (Gu and Hubschmann, 2022) was employed to generate heatmap illustrating the expression of DEGs in Sepsis.

### 2.3 Weighted gene co-expression network analysis (WGCNA)

HARGs served as the background gene set, and the ssGSEA algorithm from the GSVA package (v 1.44.5) (Gui et al., 2024) was used to calculate the HARGs score across all samples in the training set, and to compare the differences in HARGs scores between the sepsis and control groups. Based on the training set, the WGCNA package (v 1.72.5) (Langfelder and Horvath, 2008) was employed to construct a co-expression network. Prior to network construction, cluster analysis was conducted on the samples to identify and exclude outlier samples. When selecting the soft-thresholding power, we adhered to the criteria that the scale-free R^2^ value should be close to 0.9 and the mean connectivity should approach 0, ensuring that the network structure conformed to the scale-free distribution characteristic. Subsequently, a minimum of 200 genes was set for each gene module as a basis for module division. Following that, a Spearman correlation analysis was conducted between the obtained gene modules and the HARGs score of the samples, with the aim of identifying the key modules and their key module genes that had the highest absolute correlation with the HARGs scores.

### 2.4 Identification and enrichment analysis of candidate genes

The ggvenn package (v 0.1.9) (Zheng et al., 2024) was utilized to identify the intersection between DEGs and key module genes, thereby determining candidate genes. Subsequently, the clusterProfiler package (v 4.7.1.001) (Yu et al., 2012) conducted Gene Ontology (GO) and Kyoto Encyclopedia of Genes and Genomes (KEGG) enrichment analyses for these candidate genes with *P* < 0.05. The ggplot2 package was employed to visualize the enrichment results. Additionally, the STRING database (http://string.embl.de/) was used to construct a protein-protein interaction (PPI) network for the candidate genes with a confidence score of ≥0.4.

### 2.5 Selection of biomarkers

Machine learning algorithms were employed to screen candidate genes for the identification of potential biomarkers. Initially, the glmnet package (v 4.1.8) (Friedman et al., 2010) was used to perform least absolute shrinkage and selection operator (LASSO) analysis on the candidate genes, with model performance optimized through 10-fold cross-validation. Subsequently, the e1071 package (v 1.7.14) (Zhang et al., 2024) was utilized to conduct support vector machine-recursive feature elimination (SVM-RFE) analysis on the features obtained from the LASSO analysis, aiming to identify the feature genes with the lowest error rate. Finally, the Boruta package (v 8.0.0) (Liu Q. et al., 2023) was utilized to perform Boruta analysis on the feature genes derived from the SVM-RFE analysis, to screen out the final candidate biomarkers. The selection process was required to identify the final biomarkers. The pROC package (v 1.18.0) (Wang et al., 2023) was utilized to plot the receiver operating characteristic (ROC) curves for genes in the training and validation cohorts, with the aim of evaluating the diagnostic performance of candidate biomarkers. Subsequently, box plots were employed to illustrate the expression differences of genes with area under the curve (AUC) values ≥0.7 between sepsis and control samples within these two cohorts. Genes that exhibited significant and consistent trends were defined as biomarkers.

### 2.6 Reverse transcription-quantitative polymerase chain reaction (RT-qPCR) validation and function analysis of the selected biomarkers

#### 2.6.1 Cell culture and sepsis model construction

The THP-1 cell line was obtained from the American Type Culture Collection (ATCC) and cultured in RPMI-1640 medium supplemented with 10% fetal bovine serum (FBS, HyClone) and 100 U/mL penicillin-streptomycin in a 37°C incubator with 5% CO_2_. To establish an *in vitro* sepsis model, THP-1 monocytes were differentiated into macrophages by treating them with 100 nM phorbol 12-myristate 13-acetate (PMA) for 48 h. Following differentiation, the macrophages were stimulated with 1 µg/mL lipopolysaccharide (LPS) for 24 h to induce a sepsis-like inflammatory response.

#### 2.6.2 Clinical sample collection

Peripheral blood samples were collected from sepsis patients diagnosed based on Sepsis-3 criteria at The First Affiliated Hospital of Bengbu Medical University and from healthy volunteers as controls. Specifically, the diagnosis of sepsis was based on the following criteria: (Singer et al., 2016) confirmed diagnosis of sepsis based on clinical and laboratory indicators; (Su et al., 2024) age between 18 and 75 years; and (Wu et al., 2024) provision of written informed consent by the patient or legal representative. Exclusion criteria included: (Singer et al., 2016) age <18 years; (Su et al., 2024) pregnancy; (Wu et al., 2024) presence of malignant tumors; (Li et al., 2024a) autoimmune diseases; (Li et al., 2024b) recent surgery or trauma; (Sun et al., 2021) acute or chronic aseptic inflammation; (Tabone et al., 2018) chronic organ dysfunction; (Venet et al., 2017); hematological disorders; (Scicluna et al., 2015) other infections unrelated to sepsis; (van Vught et al., 2016); treatment with immunosuppressive agents; (Qiu et al., 2021) patients admitted for palliative care only; (Qin et al., 2024) existence of an advanced directive to withhold or withdraw life-sustaining treatment; or (Ritchie et al., 2015) patients or legal representatives unwilling or unable to provide informed consent. Healthy controls had no history of infection, immune-related disease, chronic illness, or medication use at the time of sample collection.

A total of 20 samples (11 sepsis and 9 healthy controls) were used for RT-qPCR validation (Tables 1, 2). Total RNA was extracted from whole blood using the TRIzol reagent following the manufacturer’s protocol. The study was approved by the hospital’s Ethics Committee, and Informed consent was obtained from all participants.

**TABLE 1:** The information of sepsis patients.

Information of patients			Blood type	Presence or absence of infection	Blood pressure (pre-hospitalization) (Systolic pressure/Diastolic pressure)/mmHg)	Diabetes	SOFA				
Bed number	Age	Gender					0	1	2	3	4
702	64	Female	—	✓	90/60	✓	MAP ≥ 70 mmHg	Oxygen index < 400 mmHg	—	Platelet Count (1 × 10^9^) < 50	—
709	51	Male	A	✓	95/61	—	MAP ≥ 70 mmHg	—	—	Oxygen index < 200&mechanical ventilation	—
701	76	Male	AB	✓	94/58	✓	MAP ≥ 70 mmHg	Bilirubin:1.2–1.9 mg/dL (20–32 μmol/L)	Oxygen index << 300 mmHg (40 kPa)	—	—
708	73	Male	O	✓	85/54	✓	—	MAP < 70 mmHg	Oxygen index < 300 mmHg (40 kPa)	Serum creatinine:3.5–4.9 mg/dL (300–400 μmol/L)	—
710	66	Female	AB	✓	82/51	✓	—	MAP < 70 mmHg	Serum creatinine:2.0–3.4 mg/dL (171–299 μmol/L)	—	—
712	85	Male	B	✓	79/51	—	—	MAP < 70 mmHg	Serum creatinine:2.0–3.4 mg/dL (171–299 μmol/L)	—	Bilirubin:> 12.0 mg/dL (204 μmol/L)
725	65	Male	B	✓	89/43	✓	—	MAP < 70 mmHg	Oxygen index < 300 mmHg (40 kPa)	Serum creatinine:3.5–4.9 mg/dL (300–400 μmol/L)&Urinary volume < 500 mL/d	—
719	51	Male	AB	✓	76/51	—	—	MAP < 70 mmHg	Serum creatinine:2.0–3.4 mg/dL (171–299 μmol/L)	Bilirubin:6.0–11.9 mg/dL (102–204 μmol/L)&Urinary volume < 500 mL/d	—
19	58	Female	O	✓	82/47	—	—	MAP < 70 mmHg	Oxygen index < 300 mmHg (40 kPa)	—	Serum creatinine> 5.0 mg/dL&Urinary volume < 200 mL/d
20	52	Male	O	✓	74/38	✓	—	MAP < 70 mmHg	Oxygen index < 300 mmHg (40 kPa)	Serum creatinine≥440&Urinary volume < 500 mL/d	Norepinephrine >0.1 µg/kg/min
17	56	Male	B	✓	78/45	✓	—	MAP < 70 mmHg	Oxygen index << 300 mmHg (40 kPa)&Serum creatinine:2.0–3.4 mg/dL (171–299 μmol/L)	Bilirubin:6.0–11.9 mg/dL (102–204 μmol/L)&Urinary volume < 500 mL/d	—

**TABLE 2:** The information of healthy people.

Number	Age	Gender	Blood type	Presence or absence of disease	Past medical history and family history of hereditary diseases
1	22	Male	O	—	—
2	23	Male	A	—	—
3	71	Female	B	—	—
4	45	Male	B	—	—
5	58	Male	AB	—	—
6	49	Male	A	—	—
7	62	Female	A	—	—
8	56	Female	O	—	—
9	46	Male	B	—	—

#### 2.6.3 RNA extraction and RT-qPCR analysis

Total RNA was extracted from LPS-stimulated THP-1 macrophages and patient blood samples using TRIzol reagent (Invitrogen, United States), according to the manufacturer’s protocol. To ensure the integrity and reliability of the collected samples, all peripheral blood specimens were processed within 2 h of collection. The concentration and purity of RNA were assessed using a NanoDrop One spectrophotometer (Thermo Fisher Scientific), ensuring an 260/280 ratio between 1.8 and 2.1. For cDNA synthesis, 500 ng of total RNA was reverse transcribed using the TransScript All-in-one First Strand cDNA Synthesis Kit (Transgenbiotech AT-341), and RT-qPCR analysis was conducted with SYBR Green-based detection (Transgenbiotech AQ-601) on a Real-Time PCR system (Applied Biosystems). The RT-qPCR conditions were initial denaturation at 94°C for 30 s, followed by 40 cycles of 94°C for 5 s and 60°C for 30 s. All reactions were performed in triplicate. Gene-specific primers (Table 3) were used for the amplification of *BLOC1S1*, *NDUFA1*, *SFT2D1*, *CXCL10*, and *IL-6*, with *GAPDH* serving as an internal control. The delta(d)CT method was employed to calculate the relative expression levels of the target genes relative to internal control gene (GAPDH) in a single sample. The ddCT method, based on the dCT calculation, was further employed to compare the relative expression changes of the target gene between the experimental group and the control group. Finally, the formula 2^−ΔΔCt^ was used to calculate the relative expression of the target gene. Data are presented as mean ± SD from at least three independent experiments (Livak and Schmittgen, 2001).

**TABLE 3:** Primer used for qPCR validation.

CXCL10	F: GTGGCATTCAAGGAGTACCTC	R: TGATGGCCTTCGATTCTGGATT
IL-6	F: TGCGCAGCTTTAAGGAGTTC	R: CCCATGCTACATTTGCCGAA
BLOC1S1	F: AGGAGGCGAGAGGCTATCAC	R: GGACCTGTAGGGTCTTCACCT
NDUFA1	F: GCGTACATCCACAGGTTCACT	R: GCGCCTATCTCTTTCCATCAGA
SFT2D1	F: GCTCTTTGGTGGCATAAGAAGG	R: GGCTATACCAGGTCATTGACAAG
GAPDH	F: GTCTCCTCTGACTTCAACAGCG	R: ACCACCCTGTTGCTGTAGCCAA

#### 2.6.4 *SFT2D1* knockdown and inflammatory cytokine assessment

To investigate the function of *SFT2D1* in sepsis, small interfering RNA (siRNA) targeting *SFT2D1*, and a scrambled siRNA control were transfected into THP-1 macrophages using Lipofectamine RNAiMAX (Invitrogen) according to the manufacturer’s protocol. After 48 h of transfection, the cells were stimulated with 1 µg/mL LPS for 24 h, followed by RNA extraction. The efficiency of *SFT2D1* knockdown was confirmed by RT-qPCR. Additionally, the mRNA expression levels of *CXCL10* and *IL-6* were assessed to evaluate the inflammatory response.

### 2.7 Construction of the nomogram

Based on the expression of biomarkers, the rms package (v 6.8–1) (Wang et al., 2024) was used to construct nomogram. To assess the accuracy of the nomogram, calibration curve was plotted. Calibration curve is an essential tool for evaluating the accuracy of predictive models, as they assess the calibration of the model by comparing predicted probabilities with actual observed outcomes. Additionally, ROC curves for the nomogram were plotted to evaluate their diagnostic performance.

### 2.8 Immune infiltration analysis

The ssGSEA algorithm from the GSVA package was employed to calculate the scores of 28 immune cell types in all samples within the training set. Histogram of immune infiltration was plotted to compare the infiltration proportions of immune cell types across different samples. Additionally, box plot was used to display the differences in the 28 immune cell types between groups. The ggcor package (v 0.9.8.1) (Ban et al., 2024) was utilized to generate a correlation heatmap to analyze the correlation between biomarkers and differential immune cells.

### 2.9 Drug prediction and molecular docking

Relying on the CoreMine database (https://coremine.com/medical/?locale=zh_CN#search), drugs associated with biomarkers were predicted. Subsequently, Cytoscape software (v 3.7.1) (Shannon et al., 2003) was employed to construct and visualize the network relationships between biomarkers and drugs. Based on the ranking of network connectivity, the drug-biomarker pairs with the highest connectivity are selected for molecular docking. The 3D structural files of drugs were obtained using the PubChem database (https://pubchem.ncbi.nlm.nih.gov/) and Babel GUI software. Concurrently, the protein 3D structures of biomarkers were downloaded from the UniProt database (https://www.uniprot.org). Thereafter, AutoDockTools software was utilized to optimize these structures and perform molecular docking. Finally, the results were visualized using PyMOL software.

### 2.10 Gene set enrichment analysis (GSEA) and gene set variation analysis (GSVA)

Utilizing the KEGG gene sets provided by the msigdbr package (v 7.5.1) (Long et al., 2023), the clusterProfiler package was employed to perform GSEA. This process was based on the expression levels of individual biomarkers and used the correlation coefficient as the basis for sorting, with |NES| > 1 and adj. *P* < 0.05. Additionally, for the GO background gene sets provided by the msigdbr package, the GSVA and limma packages were used to conduct GSVA on the sepsis and control samples in the training set, with thresholds of |t| > 2 and *P* < 0.05.

### 2.11 Construction of regulatory networks for biomarkers

The transcription factors (TFs) corresponding to the biomarkers were retrieved using the JASPAR database (https://jaspar.elixir.no/). Following this, the microRNAs (miRNAs) corresponding to the biomarkers and the long non-coding RNAs (lncRNAs) associated with these miRNAs, with clipExpNum >60, were searched in the Starbase database (https://rnasysu.com/encori/). To visually represent these complex molecular networks, the biomarker-TF and mRNA-miRNA-lncRNA networks were visualized using the software Cytoscape.

### 2.12 Mendelian randomization (MR) analysis

Utilizing biomarkers as exposure factors and sepsis as the outcome, MR analysis was conducted. Single nucleotide polymorphisms (SNPs) were employed as instrumental variables (IVs), with three primary assumptions (Singer et al., 2016): There is a significant association between SNPs and biomarkers (Su et al., 2024); SNPs are independent of potential confounding factors (Wu et al., 2024); The effect on sepsis occurs solely through the biomarkers. The genome-wide association study (GWAS) data on sepsis and expression quantitative trait locus (eQTL) data for biomarkers (Supplementary Table S4) were obtained from the IEU OpenGWAS database (https://gwas.mrcieu.ac.uk/). The sepsis dataset, ieu-b-4980, included 11,643 sepsis and 474,841 control European samples, totaling 12, 243, 539 SNPs. This study followed the STROBE-MR reporting norms (Skrivankova et al., 2021).

The TwoSampleMR package (v 0.6.3) (Chen et al., 2024) was utilized for the reading and filtering of exposure SNPs, with a stringent set for *P* < 5^10^−8^. The ld_clump() function from the ieugwasr package (v 1.0.0) (Fan et al., 2024) as employed to remove SNPs with linkage disequilibrium, with parameters set at r2 = 0.001 and kb = 10,000. Additionally, we selected IVs with an F-statistic greater than 10 to further exclude the possibility of weak IVs. Confounding factors such as “smoking status measurement”, “systolic blood pressure”, “smoking status measurement”, “diastolic blood pressure”, “smoking behavior”, “body mass index”, “type 2 diabetes mellitus”, “rheumatoid arthritis”, “cardiovascular disease”, “cardiovascular disease biomarker measurement”, “latent autoimmune diabetes in adults”, “type 2 diabetes mellitus”, “psoriasis”, “type 2 diabetes mellitus”, “smoking status measurement”, “rheumatoid arthritis”, “ACPA-positive rheumatoid arthritis”, “rheumatoid factor seropositivity measurement”, “rheumatoid arthritis”, “anti-citrullinated protein antibody seropositivity”, and “rheumatoid factor seropositivity measurement” were excluded through the GWAS catalog database (https://www.ebi.ac.uk/gwas/). The harmonise_data function from the TwoSampleMR package was used to harmonize effect alleles and effect sizes, and to exclude IVs significantly associated with the outcome. Five algorithms were employed for MR analysis, including MR Egger (Burgess and Thompson, 2017) (PMID: 28527048), weighted median (Bowden et al., 2016), inverse variance weighted (IVW) (Ding et al., 2023), simple mode (Zeng et al., 2023), and weighted mode (Zeng et al., 2023), with IVW considered the definitive analytical method. In addition, Steiger test was performed to determine the directionality of the relationship between the biomarker and sepsis.

Heterogeneity was assessed using Cochran’s Q test, with results considered reliable when the P was greater than 0.05. Additionally, the MR-Egger intercept test was used to determine horizontal pleiotropy. Finally, leave-one-out approach was utilized to identify influential outliers by sequentially removing individual SNPs and re-estimating the causal effects.

### 2.13 ScRNA-seq analysis

First, the Seurat package (v 5.1.0) (Hao et al., 2024) was utilized for preprocessing and data filtering on the GSE167363 dataset. The specific steps included removing genes detected in fewer than 200 cells and excluding cells with nFeature_RNA (the number of genes in each cell) ≥ 4,000, nCount_RNA (the total RNA count per cell) ≥ 20,000, and the proportion of mitochondrial gene expression ≥10. Subsequently, the data underwent logarithmic normalization, and the variance stabilizing transformation (vst) method was applied to select genes with high variability between cells. The top 2,000 highly variable genes (HVGs) were selected for visualization. To reduce data dimensionality, principal component analysis (PCA) was performed. The JackStrawPlot and JackStraw functions were utilized to assess the significance of the principal components (PCs), thereby selecting an appropriate number of PCs for subsequent analysis. The FindNeighbors and FindClusters functions within the Seurat package were used for unsupervised clustering of cells, with a resolution set to 0.1, and Uniform Manifold Approximation and Projection (UMAP) clustering method was applied for cell clustering. The cellmarker2.0 database (http://117.50.127.228/CellMarker/) and the literature (Zhao et al., 2023) were utilized to provide detailed annotations for the clusters obtained. Bubble plot was used to visualize the expression of biomarkers in different cell clusters, and box plots were employed to show the differences in biomarker expression across cell types between sepsis and control groups. Additionally, the monocle package (v 2.26.0) (Chen et al., 2024) was used for pseudotime analysis of cells, and the CellChat package (v 1.6.1) (Huang et al., 2024) was utilized for cell communication analysis.

### 2.14 Statistical analysis

All analyses were conducted in R version 4.4.1, with inter-group differences assessed using Wilcoxon test, establishing a significance level at *P* < 0.05. A Spearman correlation analysis was conducted between the obtained gene modules and the HARGs score of the samples. For RT-qPCR validation experiments, data are presented as mean ± standard deviation (SD) from at least three independent biological replicates. Group comparisons were performed using a multiple t-test when comparing two groups, and one-way ANOVA for multiple group comparisons. Statistical significance was defined as P < 0.05. Where applicable, P values were corrected for multiple comparisons using the Dunnett method.

## 3 Result

### 3.1 Potential functional characteristics of candidate genes

**FIGURE 1:**
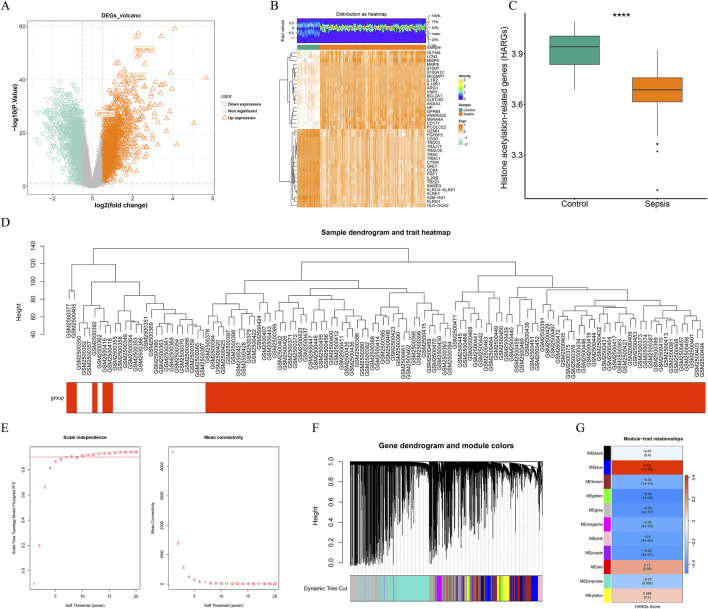
Differential expression analysis and weighted gene co-expression network analysis (WGCNA). **(A)** volcano map of differentially expressed genes (DEGs) between sepsis and control groups in the GSE95233. **(B)** heatmap of top 20 DEGs between sepsis and control samples. **(C)** histone acetylation-related genes (HARGs) score difference between sepsis and control groups. ****, *P* < 0.0001. **(D)** sample clustering tree. Red is the disease sample, white is the control sample. **(E)** the network approached the scale-free distribution when the ordinate R^2^ on the left was close to the threshold of 0.9 and the average connectivity on the right was close to 0. The optimal soft threshold was 10. **(F)** dynamic clipping tree of the 11 modules. **(G)** the heatmap showed the correlation of modules with HARGs score. MEgreen, the module with the highest absolute correlation with HARGs score, was selected as the key module, with a total of 763 genes.

**FIGURE 2:**
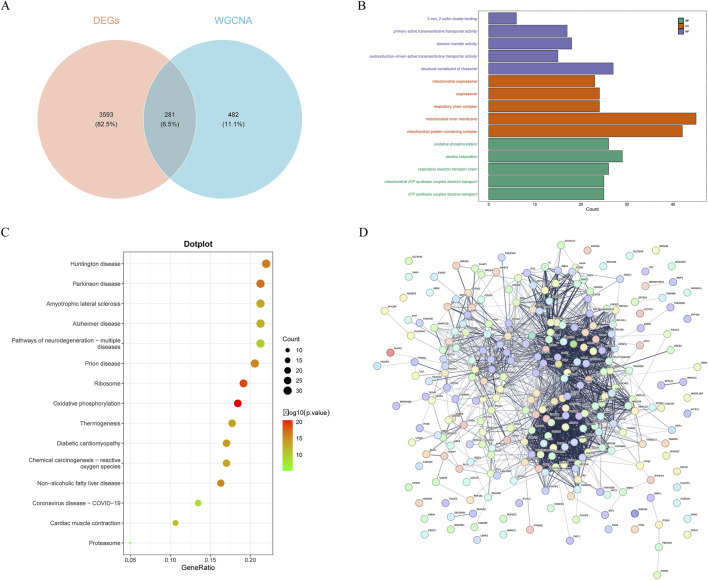
Screening and protein-protein interaction (PPI) network of candidate genes. **(A)** the intersection of DEGs, key module genes, HARGs resulted in 281 candidate genes. **(B)** Gene ontology (GO) enrichment analysis of candidate genes. Biological process, BP; cellular component, CC; molecular function, MF. **(C)** Kyoto Encyclopedia of Genes and Genomes (KEGG) enrichment analysis of candidate genes. **(D)** PPI network of candidate genes.

A total of 3,874 DEGs between sepsis and control samples were identified from the training cohort, of which 2,114 were upregulated while 1,760 downregulated (Figures 1A,B)Prior to performing WGCNA, differential analysis of the HARGs score was conducted between the sepsis and control groups. The results revealed a significant difference (*P* < 0.05) between the two groups, indicating that the HARGs score was a valid phenotypic indicator suitable for subsequent analysis (Figure 1C). Cluster analysis of all samples confirmed the absence of outlier samples (Figure 1D). The optimal soft-thresholding power for achieving a scale-free network distribution was determined to be 10 (Figure 1E). And based on this, a minimum of 200 genes per gene module was set, ultimately identifying 11 gene modules (Figure 1F). Among these modules, the MEgreen module (cor = −0.48, *P* < 0.001) showed the highest correlation with the HARGs score and was thus identified as the key module, encompassing 763 key module genes (Figure 1G). By performing an intersection analysis between DEGs and key module genes, 281 potential candidate genes were filtered out (Figure 2A).

Enrichment analysis can identify specific features or functions that are significantly enriched from a large amount of data, aiding in the understanding of the functions of genes within cells, the biological processes they participate in, and their interrelationships. Therefore, enrichment analysis on these candidate genes was conducted. The GO enrichment analysis yielded 332 entries, with 186 significantly enriched in biological processes, 95 enriched in cellular components, and 51 enriched in molecular functions (Figure 2B). Histone modification-related terms such as “histone exchange”, “negative regulation of protein modification by small protein conjugation or removal”, and “regulation of protein modification by small protein conjugation or removal” were significantly enriched, suggesting that the candidate genes might play a key role in regulating gene expression and chromatin structure. Additionally, mitochondrial-related pathways also showed enrichment, including “mitochondrial ATP synthesis coupled electron transport”, “mitochondrial protein-containing complex”, “mitochondrial inner membrane”, and “mitochondrial respirasome”, emphasizing the potential role of candidate genes in mitochondrial function and energy metabolism. KEGG pathway enrichment analysis identified 24 significantly enriched pathways, mainly enriched in “proteasome”, “protein processing in endoplasmic reticulum”, and “ribosome” (Figure 2C). These pathways were closely related to protein degradation, processing, and synthesis, further confirming the central role of candidate genes in protein metabolism.

To gain a deeper understanding of the functions of the candidate genes and their mechanisms of interaction, PPI network was constructed for the candidate genes. This network comprised 265 nodes and 2,130 PPI pairs (Figure 2D). It was evident that multiple genes had close relationships with other genes, reflecting their complex interactions within the organism. For instance, genes like *RPS24*, *COX5B*, and *ETFA* showed a high degree of interactivity in the network, which might imply their significant role in cellular functions.

### 3.2 Identification of *BLOC1S1*, *NDUFA1*, and *SFT2D1* as biomarkers through machine learning

**FIGURE 3:**
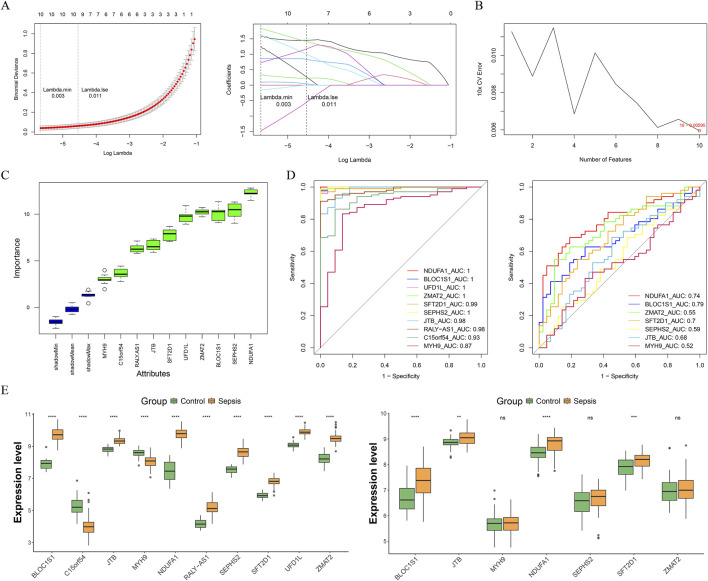
Acquisition of biomarkers. **(A)** least absolute shrinkage and selection operator (LASSO) regression analysis was performed for 10-fold cross-validation of the screened genes to obtain 10 characterized genes. **(B)** support vector machine-recursive feature elimination (SVM-RFE) analysis on the features obtained from the LASSO analysis. **(C)** Boruta agorithm screens for candidate biomarkers from the SVM-RFE analysis. Green represents important feature. **(D)** receiver operating characteristic (ROC) curves of candidate biomarkers in the training and validation sets. Genes with area under the curve (AUC) values ≥0.7 were screened to obtain *BLOC1S1*, *NDUFA1*, and *SFT2D1*. **(E)** expression box maps of candidate biomarkers between control and sepsis samples were used in the training dataset (left) and validation dataset (right) to screen for genes with exhibited significant and consistent trends. ***, *P* < 0.001; ****, *P* < 0.0001.

With the advancement of machine learning techniques, we were capable of more accurately identifying genes of significant biological importance from complex data sets. Leveraging this, we employed a stepwise machine learning approach to screen out more critical genes from a pool of candidates. Through LASSO regression analysis, we successfully identified 10 genes (Lambda.min = 0.003), including *NDUFA1*, *BLOC1S1*, *UFD1L*, *ZMAT2*, *SFT2D1*, *SEPHS2*, *JTB*, *RALY-AS1*, *C15orf54*, and *MYH9* (Figure 3A). Subsequently, SVM-RFE analysis was conducted on these 10 genes, and at a minimum error rate of 0.00595, all 10 genes were confirmed as feature genes (Figure 3B). Furthermore, Boruta analysis reaffirmed these 10 genes as important feature genes (Figure 3C). Thus, these 10 genes were considered candidate biomarkers. Cross-validation through multiple methods was employed to screen for biomarkers. Initially, *BLOC1S1*, *NDUFA1*, and *SFT2D1* demonstrated AUC values of ≥0.70 in both the training and validation sets, indicating good diagnostic efficacy (Figure 3D). Subsequently, the expression levels of these 10 genes in sepsis and control samples were analyzed in the training and validation sets, and the results showed that *BLOC1S1*, *NDUFA1*, and *SFT2D1* were significantly different in the training and validation sets, and the AUC values for these three genes were ≥0.70 (*P* < 0.001) (Figure 3E). Therefore, *BLOC1S1*, *NDUFA1*, and *SFT2D1* were identified as the final biomarkers.

### 3.3 Characterizing the function of biomarkers and sepsis

**FIGURE 4:**
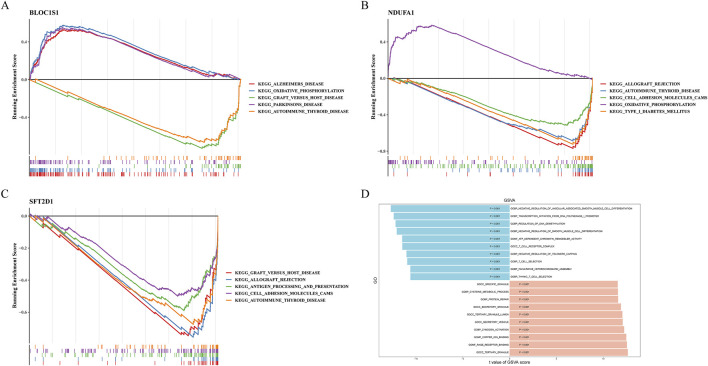
Gene set enrichment analysis (GSEA) and gene set variation analysis (GSVA). **(A–C)** GSEA of *BLOC1S1*, *NDUFA1*, and *SFT2D1*, respectively. **(D)** compare with control group, GSVA of sepsis group.

GSEA provides a global perspective to reveal overall patterns of biological processes in gene expression data. Based on this, GSEA was performed on the biomarkers to uncover gene expression patterns associated with specific biological pathways (Figures 4A–C). The results showed that multiple pathways, including “oxidative phosphorylation”, “antigen processing and presentation”, “proteasome”, “ribosome”, and “FC gamma R-mediated phagocytosis”, were significantly enriched *in BLOC1S1*, *NDUFA1*, and *SFT2D1*. These findings indicated that the biomarkers played a significant role in energy metabolism, immune response, and protein homeostasis. Additionally, the “toll-like receptor signaling pathway” was activated *in BLOC1S1* and *SFT2D1*, which might indicate a key regulatory mechanism of the inflammatory response in sepsis.

GSVA can reveal changes in biological processes and signal pathways by assessing the activity changes of predefined gene sets in different samples. Using this method, we explored pathways related to sepsis (Figure 4D). The GSVA results further revealed that T cell-related pathways were suppressed in sepsis, including “thymic T cell selection”, “T cell selection”, and “T cell receptor complex”. The suppression of these pathways might be associated with abnormal T cell function in sepsis, affecting the immune response. Moreover, pathways related to regulation, such as “negative regulation of telomere capping” and “regulation of DNA demethylation”, were also suppressed. The suppression of these pathways might be related to abnormalities in cellular senescence and epigenetic regulation. In contrast, “RAGE receptor binding”, “protein repair”, and “secretory vesicle” were activated in sepsis. The activation of these pathways might be related to the inflammatory response, cellular damage, and repair mechanisms in sepsis.

### 3.4 Nomogram for predicting sepsis risk

**FIGURE 5:**
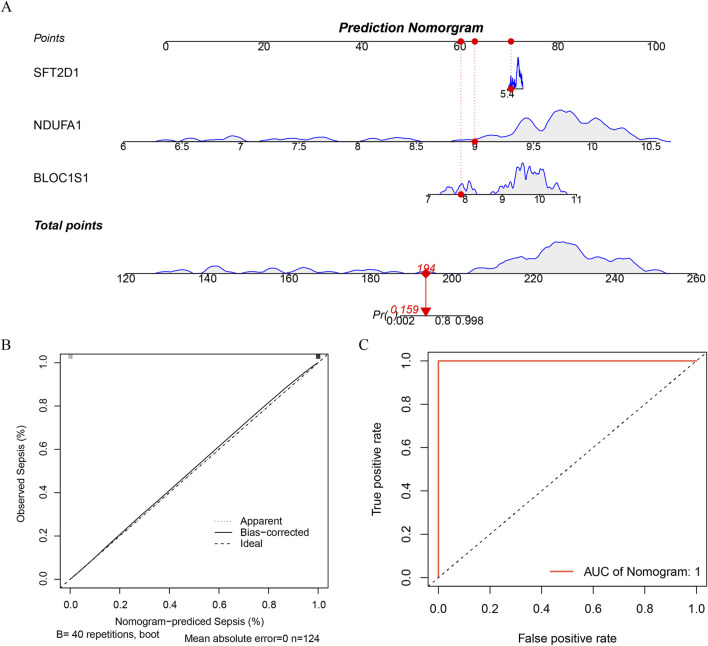
Construction of a nomogram. **(A)** construct a nomogram based on the expression of each biomarker. **(B)** calibration curve of nomogram. **(C)** ROC curve of nomogram.

Nomogram, as an intuitive predictive tool, can integrate multiple biomarkers to forecast an individual’s risk of disease. By incorporating the three identified biomarkers *BLOC1S1*, *NDUFA1*, and *SFT2D1* into the nomogram model, we have established a comprehensive diagnostic tool (Figure 5A). To ensure the predictive accuracy of the nomogram, calibration curve was employed for validation. The slope of the calibration curve being close to one indicated that our nomogram model was highly accurate in predicting disease risk (Figure 5B). Furthermore, the diagnostic efficacy of the model was assessed using the ROC curve. The AUC value of the nomogram reached 1, which, while indicating that the model has extremely high efficacy in distinguishing between diseased and non-diseased individuals, also suggested a potential for overfitting (Figure 5C). Nonetheless, the model’s effectiveness in disease diagnosis remained significant, providing clinicians with a powerful tool to aid in making more accurate diagnostic decisions. This offered a new perspective for the early diagnosis of the disease and personalized treatment.

### 3.5 Immune cells infiltration of biomarkers in sepsis

**FIGURE 6:**
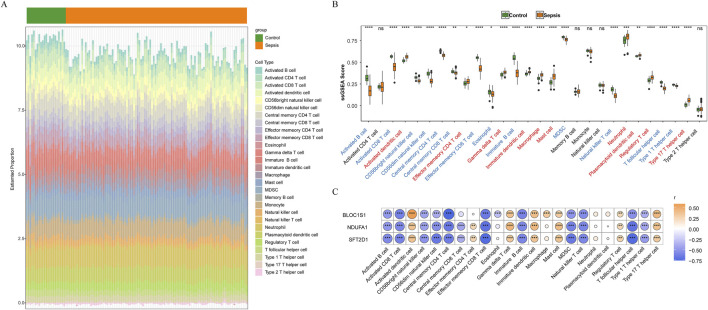
Immune infiltration analysis. **(A)** stacked bar chart of immune cell score between sepsis and control samples. **(B)** box plot of immune cell infiltration between sepsis and control samples. ns, no significance; *, *P* < 0.05; **, *P* < 0.01; ***, *P* < 0.001; ****, *P* < 0.0001. **(C)** correlation between biomarkers and immune cells. *, *P* < 0.05; **, *P* < 0.01; ***, *P* < 0.001; ****, *P* < 0.0001.

Immune cells play a pivotal role in the complex physiological dysregulation triggered by sepsis, with their behavior changes being directly associated with disease progression (Wang et al., 2022). Through immune infiltration analysis, we explored the immunological alterations in sepsis. In addition, the histogram of immune infiltration was constructed to visually display the differences in immune cell scores between sepsis and control samples (Figure 6A). Notably, there was a significant difference in the scores of 23 immune cell types between groups (Figure 6B). Activated B cells and Activated CD8 T+ cells showed lower infiltration levels in sepsis, while activated dendritic cells, neutrophils, and type 17 T helper cells exhibited higher infiltration levels in sepsis. These findings revealed the activation status and functional changes of specific immune cells in sepsis. The correlation between biomarkers and immune cells was analyzed to explore the role of biomarkers in immune infiltration (Figure 6C). Among them, *BLOC1S1* showed the strongest positive correlation with activated dendritic cells (cor = 0.577, *P* < 0.001), suggesting that *BLOC1S1* might be involved in the regulation of activated dendritic cell processes, thereby affecting the immune response in sepsis. Concurrently, *BLOC1S1* exhibited the strongest negative correlation with central memory CD4^+^ T cells (cor = −0.761, *P* < 0.001), indicating that *BLOC1S1* might suppress the function or quantity of central memory CD4^+^ T cells, playing a complex role in the immunological dysregulation of sepsis.

### 3.6 Complex regulatory networks of biomarkers

**FIGURE 7:**
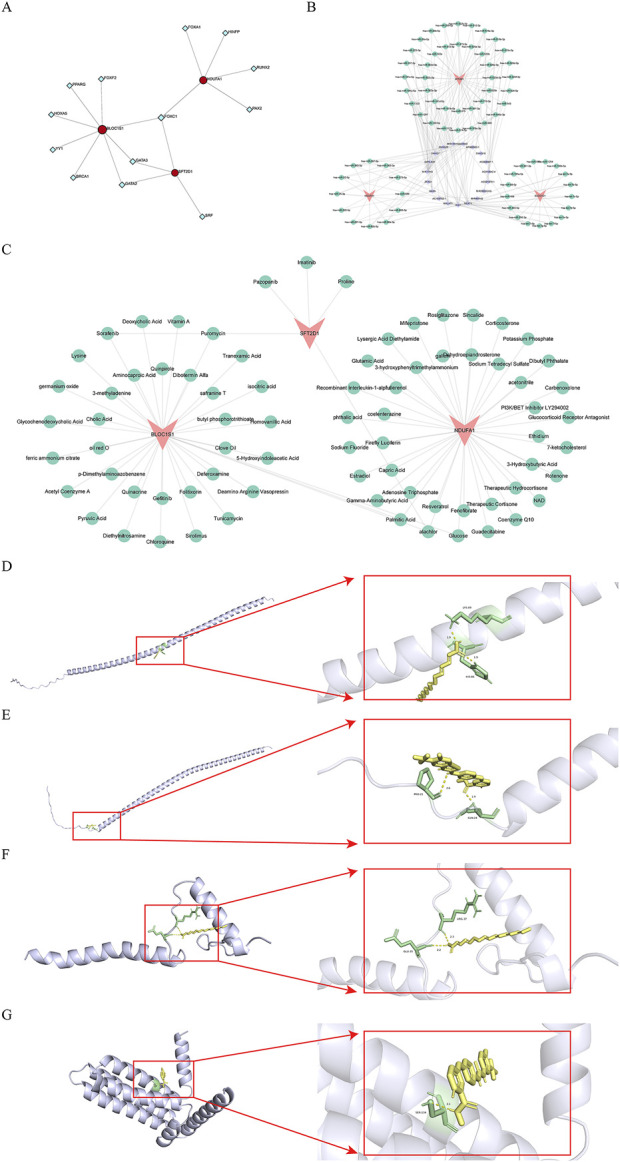
Construction of regulatory network, drug predition, and molecular docking. **(A)** the network of transcription factors (TFs)-biomarkers. Red is biomarkers, blue is TFs. **(B)** the network of mRNA-microRNA (miRNA)-long non-coding RNA (lncRNA). Orange is biomarkers, purple is the lncRNA, and green is the miRNA. **(C)** the identification of drugs targeting biomarkers. Pink is biomarkers, green is drugs. **(D,E)** molecular docking mode diagram of *BLOC1S1* with Palmitic Acid and Sorafenib, respectively. **(F)** molecular docking mode diagram of *NDUFA1* with Palmitic Acid. **(G)** molecular docking mode diagram of *SFT2D1* with Sorafenib.

TFs play a crucial role in regulating gene transcription. Through the prediction of TFs for biomarkers, we identified a total of 13TFs and 17 interaction pairs. Notably, FOXC1 was concurrently predicted in *BLOC1S1*, *NDUFA1*, and *SFT2D1*, while GATA3 and GATA2 were predicted in both *BLOC1S1* and *SFT2D1* (Figure 7A). These results suggested that these TFs might play a central role in regulating the expression of these biomarkers. Furthermore, a comprehensive dissection of the gene expression regulatory network was conducted. Prediction of miRNAs for biomarkers yielded 69 miRNAs corresponding to three biomarkers. Subsequently, prediction of lncRNAs corresponding to miRNAs resulted in 19 lncRNAs corresponding to 59 miRNAs. The construction of an mRNA-miRNA-lncRNA network predicted a total of 69 miRNAs and 19 lncRNAs, with a total of 186 interaction pairs (Figure 7B). This complex network revealed the multi-level regulation of biomarkers, where MALAT1 indirectly regulated *BLOC1S1* and *SFT2D1* by modulating hsa-miR-498. Multiple lncRNAs simultaneously regulated specific miRNAs to indirectly regulate biomarkers, such as OIP5-AS1, XIST, NORAD regulating hsa-miR-32–5p to indirectly affect *NDUFA1*. Additionally, specific lncRNAs regulated multiple miRNAs to indirectly regulate biomarkers, including XIST regulating hsa-let-7a-5p, hsa-let-7i-5p, hsa-let-7f-5p to indirectly affect *BLOC1S1*. These findings enriched our understanding of the regulatory network of biomarkers.

### 3.7 Potential therapeutic strategies for sepsis

The identification of potential drugs targeting biomarkers is crucial for the development of personalized treatment plans. A biomarker-drug network was constructed, which included three biomarkers, 76 drugs, and 81 interaction pairs (Figure 7C). Notably, sorafenib was concurrently predicted in both *BLOC1S1* and *SFT2D1*, while alachlor was concurrently predicted in *BLOC1S1* and *NDUFA1*. These findings suggested that these drugs might play a significant role. To further determine the binding capacity between biomarkers and drug targets, molecular docking was performed. The docking binding energies for *BLOC1S1* with palmitic acid and sorafenib were −2.8 and −6.15 kcal/mol, respectively; for *NDUFA1* with palmitic acid, it was −5.57 kcal/mol; and for *SFT2D1* with sorafenib, it was −4 kcal/mol. These negative values indicated a strong binding affinity. Subsequently, the binding modes were visualized, with *BLOC1S1* forming hydrogen bonds with residues LYS-89, HIS-86 of palmitic acid and residues PRO-21, GLN-24 of sorafenib (Figures 7D,E); *NDUFA1* forming hydrogen bonds with residues GLU-35, ARG-37 of palmitic acid (Figure 7F). And *SFT2D1* forming a hydrogen bond with residue SER-134 of sorafenib (Figure 7G). These findings provided important clues for drug development.

### 3.8 *SFT2D1* as a risk factor for sepsis

To explore the causal relationship between *BLOC1S1*, *NDUFA1*, and *SFT2D1* and sepsis, MR analysis was conducted. We included 280 SNPs for analysis, with the F-values ranging from 30.003 to 4717.202 (Supplementary Table S5). The IVW results indicated a causal link, identifying *SFT2D1* as a risk factor for sepsis [odds ratio (OR) = 1.070, 95% confidence interval (CI) = 1.016–1.127, *P* = 0.001] (Table 4). The forest plot further showed that the effect size of *SFT2D1* on sepsis was overall greater than 0 (Supplementary Figure S1A). A scatter plot demonstrated a positive correlation between *SFT2D1* expression and increased risk of sepsis (Supplementary Figure S1B). To test the directionality of the MR analysis, a Steiger directionality test was performed (Table 5). The results confirmed the correct direction of the analysis for *SFT2D1*, with no evidence of bidirectional relationships. Sensitivity analyses further validated the accuracy of the MR analysis. Specifically, Cochran’s Q test and pleiotropy tests showed no evidence of heterogeneity and confounding bias (*P* > 0.05), indicating that our analysis results were robust (Table 6). The funnel plot revealed a roughly symmetrical distribution on both sides (Supplementary Figure S1C). Leave-one-out analysis showed that sequentially excluding each SNP had minimal impact on the results, further confirming the robustness of the findings (Supplementary Figure S1D). However, *BLOC1S1* and *NDUFA1* did not screen suitable SNP, so MR analysis was not performed.

**TABLE 4:** Results of Mendelian randomization (MR) analysis of SFT2D1 and sepsis.

Exposure	Outcome	Method	nsnp	se	Pval	Or	or_lci95	or_uci95
SFT2D1	Sepsis	MR Egger	3	0.146	0.907	0.979	0.736	1.302
		Weighted median	3	0.027	0.014	1.069	1.014	1.127
		Inverse variance weighted	3	0.026	0.010	1.070	1.016	1.127
		Simple mode	3	0.032	0.163	1.073	1.007	1.143
		Weighted mode	3	0.028	0.146	1.067	1.010	1.128

Note: nsnp, number of single nucleotide polymorphism; pval, pvalue; or, odds ratio; CI, confidence interval.

**TABLE 5:** Results of Steiger test.

Exposure	Outcome	snp_r2.exposure	snp_r2.outcome	correct_causal_direction	steiger_pval
SFT2D1	Sepsis	0.1161	1.47E-05	TRUE	0

Note: snp, single nucleotide polymorphism; pval, pvalue.

**TABLE 6:** Heterogeneity and horizontal pleiotropy tests by MR analysis.

Exposure	Outcome	Method	Q_df	Q_pval	se	Pval
SFT2D1	Sepsis	MR Egger	1	0.751		
		IVW	2	0.783	0.058	0.645

Note: Q, Cochran’s Q heterogeneity statistic; df, degree of freedom; se, standard error; pval, pvalue.

### 3.9 Exploring the developmental trajectory and communication network of macrophages

**FIGURE 8:**
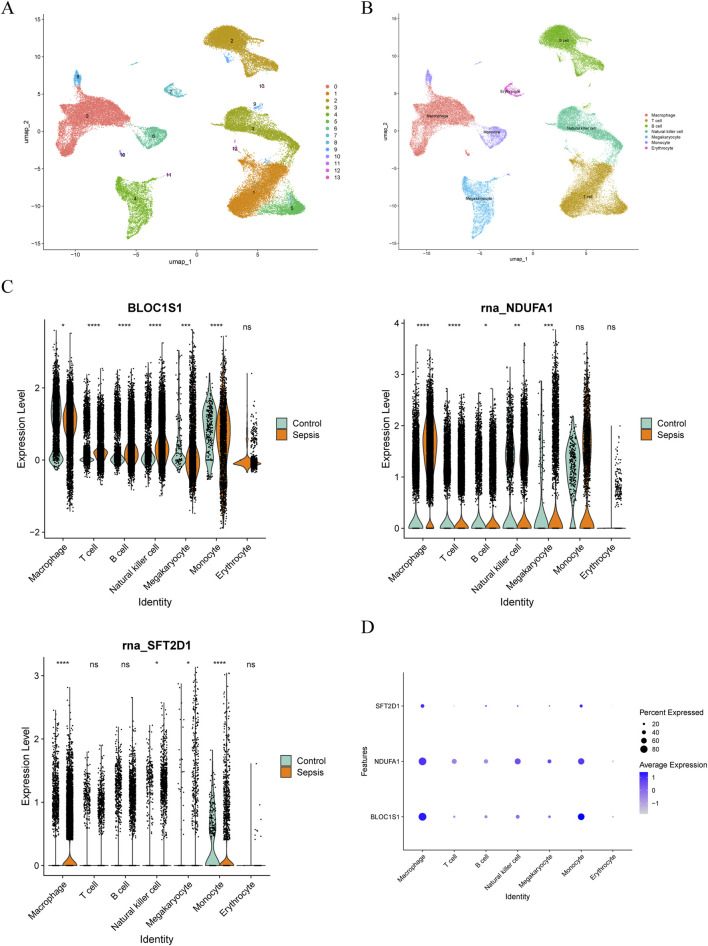
Single-cell RNA sequencing (scRNA-seq) in GSE167363. **(A)** cellular Uniform Manifold Approximation and Projection (UMAP) clustering map with 14 cell clusters. **(B)** annotation to seven cell types. **(C)** box plot of the expression of biomarker in cell types between sepsis and control groups. Ns, no significance; *, *P* < 0.05; **, *P* < 0.01; ***, *P* < 0.001; ****, *P* < 0.0001. **(D)** bubble plot of biomarkers expression in various cell types.

**FIGURE 9:**
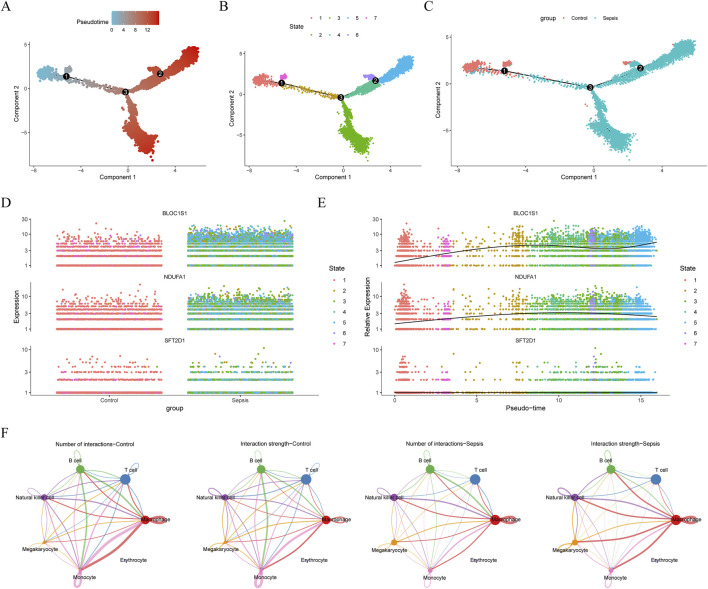
Pseudotime and cell communication analysis of macrophages. **(A)** time trajectory differentiation of macrophages. **(B)** macrophage differentiation was divided into seven stages. **(C)** macrophage cell differentiation locus in sepsis and control groups. **(D)** expression distribution of biomarkers in a macrophage sample. **(E)** expression distribution of biomarkers at different differentiation stages in macrophage. **(F)** cell communication analysis between sepsis and control samples. ns, no significance; *, *P* < 0.05; **, *P* < 0.01; ***, *P* < 0.001; ****, *P* < 0.0001.

ScRNA-seq analysis technology enables the tracking of cellular lineages and destinies throughout development and disease processes. Utilizing this technology, we have explored the expression regulation of biomarkers at the single-cell level. After rigorous data filtering, we obtained a total of 50,690 cells and 23,025 genes (Supplementary Figure S2A). For in-depth analysis, the top 2,000 HVGs and the top 30 PCs were selected for subsequent analysis (Supplementary Figure S2B–C). Through PCA dimensionality reduction and clustering analysis, we successfully divided the cells into 14 distinct clusters (Figure 8A) and annotated seven cell types, including macrophage, T cell, monocyte, natural killer cell, B cell, megakaryocyte, and erythrocyte (Figure 8B). Analysis of the expression of biomarkers across various cell types revealed significant differences in macrophage, natural killer cell, and megakaryocyte among different groups (Figure 8C). Notably, in macrophages, the expression distribution of these biomarkers was more abundant (Figure 8D), leading us to select macrophages as key cells for further in-depth analysis. Trajectory analysis can infer the chronological order of cell development or changes, revealing the evolutionary paths and critical turning points of cells in different states. By capturing patterns of gene expression changes, we reconstructed the developmental trajectories or dynamic processes of cells. By arranging macrophages in chronological order, we demonstrated the dynamic changes of cells over time and divided them into seven stages (Figures 9A,B). We found that the control group had more differentiation in state one and 7, concentrated in the early stages of differentiation, while the sepsis group was present in all stages of macrophage differentiation (Figure 9C). Further exploration of the expression of biomarkers along the temporal trajectory revealed that *BLOC1S1*, *NDUFA1*, and *SFT2D1* were mainly concentrated in state one of macrophages in the control group. In the sepsis group, these biomarkers were more abundant during differentiation stages state 3, 4, and 5 of macrophages (Figure 9D). Analysis of the expression trends of biomarkers during the differentiation process of macrophages showed that the expression of *BLOC1S1* and *NDUFA1* increased with cell differentiation, while the expression of *SFT2D1* did not change significantly (Figure 9E). These results provided us with dynamic regulatory information about these biomarkers during the differentiation process of macrophages. Cell communication analysis, by deeply parsing the interactions and regulatory networks between cells, reveals the coordination mechanisms between cells in life activities. Compared with the control group, the number and strength of interactions between macrophages and monocytes in the sepsis group were reduced, which might be part of the immunosuppressive characteristics of sepsis (Figure 9F). In contrast, the interaction strength between macrophages and B cells increased, suggesting that macrophages might promote antibody production and the formation of immune memory by enhancing interactions with B cells.

### 3.10 Experimental validation and functional analysis of acetylation-related genes in sepsis

**FIGURE 10:**
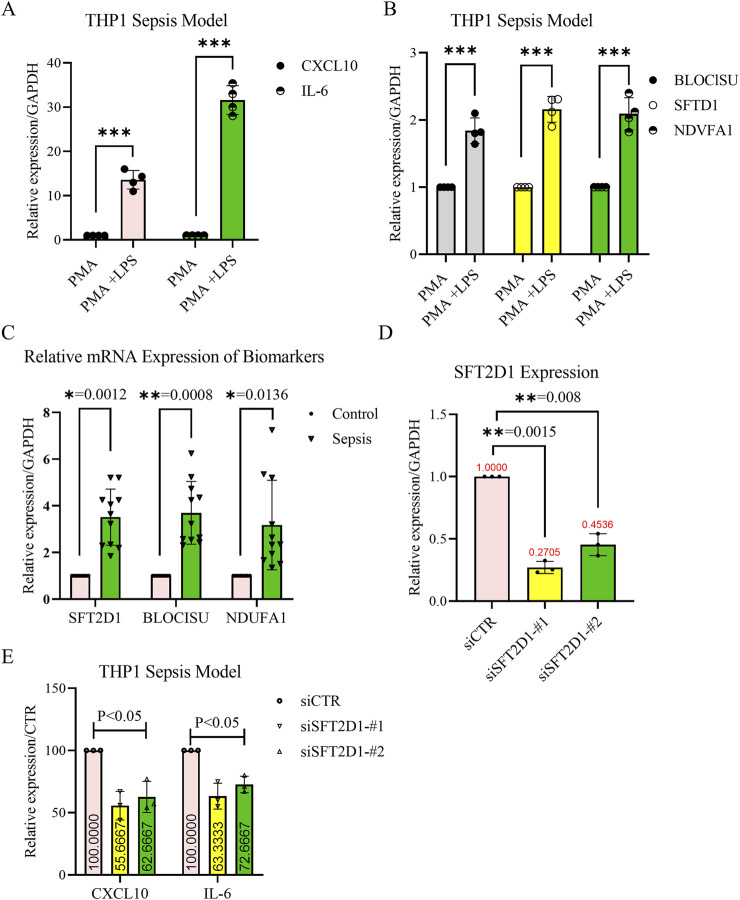
Validation of biomarker expression and functional analysis of *SFT2D1* in sepsis. **(A,B)** Reverse transcription-quantitative polymerase chain reaction (RT-qPCR) confirmation of the THP-1-derived sepsis model. THP-1 monocytes were differentiated into macrophages using PMA (100 nM, 48 h) and then stimulated with LPS (1 μg/mL, 24 h). The relative mRNA expression levels of *CXCL10*, *IL-6*, *BLOC1S1*, *NDUFA1*, and *SFT2D1* were significantly upregulated in LPS-treated cells compared to untreated controls. GAPDH was used as an internal control. (n = 4 independent experiments, mean ± SD, **P < 0.01). **(C)** RT-qPCR validation of biomarker expression in clinical blood samples. Peripheral blood was collected from sepsis patients and healthy controls. The expression levels of *BLOC1S1*, *NDUFA1*, and *SFT2D1* were significantly higher in sepsis patients compared to healthy controls (n = 9 for healthy and n = 11 for sepsis group, mean ± SD, **P < 0.01). **(D)** Knockdown efficiency of *SFT2D1* in THP-1-derived macrophages. THP-1 macrophages were transfected with siRNA targeting *SFT2D1* or scrambled siRNA as a control. RT-qPCR analysis confirmed a significant reduction in *SFT2D1* expression after siRNA transfection (n = 3 independent experiments, **P < 0.05). **(E)** Effect of *SFT2D1* inhibition on inflammatory cytokines. After *SFT2D1* knockdown, *CXCL10* and *IL-6* mRNA levels were measured by RT-qPCR. *SFT2D1* suppression significantly reduced the expression of both pro-inflammatory cytokines in LPS-stimulated macrophages, indicating its role in promoting sepsis-related inflammation (n = 3 independent experiments, **P < 0.05). (Statistical significance was determined using multiple t-tests for two-group comparisons and one-way ANOVA followed by Dunnett’s *post hoc* test for multiple group comparisons; *P* < 0.05; *P* < 0.01; *P* < 0.001. Error bars represent mean ± SD.).

To validate the expression levels of the identified acetylation-related biomarker genes *BLOC1S1*, *NDUFA1*, and *SFT2D1*, we established a sepsis model in THP-1-derived macrophages by treating cells with phorbol 12-myristate 13-acetate (PMA) for differentiation, followed by lipopolysaccharide (LPS) stimulation (Figure 10A). RT-qPCR analysis revealed that the expression of all three genes was significantly upregulated in LPS-treated macrophages compared to controls (*P* < 0.01) (Figure 10B), supporting their potential roles in sepsis pathophysiology. Furthermore, validation using patient blood samples confirmed the elevated expression levels of *BLOC1S1*, *NDUFA1*, and *SFT2D1* in sepsis patients compared to healthy controls (Figure 10C). Consistent with our bioinformatics analysis. These findings further reinforced the reliability of these genes as potential biomarkers for sepsis diagnosis and prognosis.

To assess the functional role of *SFT2D1*, which was identified as a genetic risk factor for sepsis through MR analysis, we conducted gene knockdown experiments in the LPS-induced THP-1 sepsis model. siRNA-mediated suppression of *SFT2D1* resulted in a significant reduction in its mRNA expression(Figure 10D) confirming effective knockdown. Further analysis demonstrated that *SFT2D1* inhibition significantly decreased the expression levels of key inflammatory cytokines *CXCL10* and *IL-6* (*P <* 0.05) (Figure 10E), suggesting a role for *SFT2D1* in promoting the inflammatory response in sepsis. These results indicate that targeting *SFT2D1* may have potential therapeutic implications for mitigating excessive inflammation in sepsis.

Taken together, these findings provide robust experimental evidence that the acetylation-related biomarkers identified in this study play a significant role in sepsis progression. Moreover, *SFT2D1* inhibition effectively attenuates inflammatory cytokine expression, highlighting its potential as a novel therapeutic target for sepsis management.

## 4 Discussion

Sepsis is a life-threatening condition characterized by a dysregulated host immune response to infection, resulting in systemic inflammation and organ dysfunction (Meziani et al., 2024). Despite advances in understanding its pathophysiology, the identification of reliable biomarkers for early diagnosis and treatment remains a challenge. This study identified three histone acetylation-related genes, *BLOC1S1*, *NDUFA1*, and *SFT2D1*, as potential biomarkers for sepsis through integrated bioinformatics analysis. These findings were validated in THP-1 cell-derived sepsis models and clinical blood samples, confirming their elevated expression in sepsis. Furthermore, functional experiments demonstrated that suppression of *SFT2D1* significantly reduced the expression of inflammatory cytokines *CXCL10* and *IL-6*, suggesting its potential role in modulating the inflammatory response in sepsis.

### 4.1 Biomarker function and enrichment analysis

The identified biomarkers are significantly enriched in pathways related to energy metabolism, immune response, and protein homeostasis, such as oxidative phosphorylation, proteasome activity, and ribosome function. For instance, mitochondrial dysfunction, a hallmark of sepsis, disrupts oxidative phosphorylation (Pham et al., 2024), impairing immune cell activity and energy production. Previous studies have linked mitochondrial dysfunction with decreased immune responses in sepsis (Li et al., 2024c; Zou et al., 2024; Han et al., 2025), aligning with our findings of enriched mitochondrial pathways.

*BLOC1S1*, also known as *GCN5L1*, is involved in acetyl-CoA binding and mitochondrial protein acetylation, regulating mitochondrial respiration and ATP synthesis (Sharma et al., 2024). Previous studies identified *BLOC1S1* as a critical gene associated with sepsis outcomes, consistent with its role in this study (Lai et al., 2022). *NDUFA1*, a component of mitochondrial complex I, plays a pivotal role in electron transport and energy production, highlighting its involvement in sepsis-related metabolic dysregulation (Tian et al., 2024). *SFT2D1*, though less studied, was confirmed as a risk factor for sepsis through MR analysis, highlighting its potential role in disease progression. Our experimental findings further demonstrated that *SFT2D1* suppression mitigates pro-inflammatory cytokine expression, suggesting a functional role in sepsis pathogenesis. In the present study, GSEA revealed that *SFT2D1* may exert a crucial regulatory role in the inflammatory response of sepsis by activating the toll-like receptor signaling pathway. Studies have shown that the upregulation of the toll-like receptor 2 (TLR2) pathway in keratinocytes enhances the expression of proinflammatory cytokines and chemokines, such as *IL-8*, *IL-1*β, *TNF-*α, *CCL5*, *CXCL9*, Chemokine C-X-C ligand 10 (*CXCL10*), and *CXCL11* (Yang et al., 2024). *CXCL10* is a pro-inflammatory cytokine that promotes the recruitment and activation of immune cells to infected areas (Kariminik et al., 2016). In addition, it has been shown that toll-like receptors (TLRs) promote interleukin-6 (*IL-6*) secretion (Xie et al., 2017). And *IL-6* is a pleiotropic pro-inflammatory cytokine (Kaur et al., 2020). Therefore, *SFT2D1* may indirectly affects the expression levels of *CXCL10* and *IL-6* by activating the toll-like receptor signaling pathway.

Clinically, BLOC1S1, NDUFA1, and SFT2D1—have also been implicated in various disease contexts beyond sepsis. For instance, BLOC1S1 has been associated with mitochondrial dysfunction and metabolic regulation in hepatocellular carcinoma, where it plays a role in oxidative phosphorylation and cellular metabolism (Han et al., 2023). NDUFA1, a component of mitochondrial complex I, has similarly been linked to neurodegenerative diseases and multiple tumor types (Yin et al., 2025; Kim et al., 2017), reflecting its central role in energy metabolism. Although less extensively studied, SFT2D1 has recently attracted attention in oncology. In cervical cancer, it was identified as an independent risk factor promote angiogenesis, immune suppression, and tumor cell proliferation (Kang et al., 2024). Furthermore, alternative splicing events of SFT2D1 have been significantly associated with increased pancreatic cancer risk (Liu D. et al., 2023), suggesting a broader role in tumorigenesis.

### 4.2 Immune dysregulation and biomarker impact

Immune cell infiltration analysis revealed significant correlations between the biomarkers and specific immune cell types, highlighting their roles in immune dysregulation during sepsis. *BLOC1S1* was positively correlated with activated dendritic cells, suggesting its involvement in antigen presentation and immune activation. Conversely, its negative correlation with central memory CD4^+^ T cells indicates a potential suppressive effect on adaptive immunity. These findings reflect the dual roles of immune activation and suppression in sepsis, which contribute to disease progression and poor outcomes.

Furthermore, scRNA-seq analysis confirmed that these biomarkers are highly expressed in macrophages, which play a central role in immune response for sepsis. Macrophage differentiation trajectories revealed increased expression of *BLOC1S1* and *NDUFA1* during later stages of sepsis, suggesting their involvement in immune cell reprogramming. Additionally, cell communication analysis highlighted altered interactions between macrophages and other immune cells, reflecting the complex immune dysregulation in sepsis. Our functional experiments support this notion, as *SFT2D1* knockdown in macrophages led to a reduction in pro-inflammatory cytokines, providing direct evidence for its role in inflammation regulation.

### 4.3 Therapeutic implications

Beyond biomarker identification, our study explored the therapeutic potential of targeting these genes. Drug-biomarker network analysis identified sorafenib and palmitic acid as potential therapeutic agents for *BLOC1S1*, *NDUFA1*, and *SFT2D1*. Molecular docking studies demonstrated strong binding affinities between these compounds and the biomarkers, suggesting possible mechanisms for therapeutic intervention. Importantly, our experimental results highlight the potential of *SFT2D1* inhibition in reducing inflammatory responses, providing a new avenue for therapeutic development in sepsis management.

### 4.4 Study strengths and limitations

This study integrated bioinformatics, RT-qPCR validation, Mendelian randomization, and functional experiments to identify and characterize acetylation-related biomarkers in sepsis. The experimental validation of these biomarkers in both THP-1 macrophages and clinical samples strengthens the reliability of our findings. Additionally, functional inhibition of *SFT2D1* provides direct mechanistic insight into its role in sepsis-associated inflammation.

However, some limitations should be considered. First, while RT-qPCR validation confirmed biomarker expression in sepsis, additional protein-level validation (e.g., Western blot, ELISA) would further substantiate these findings. Second, the molecular mechanisms linking *SFT2D1* to inflammation remain unclear, necessitating further studies on its regulatory pathways. Third, while our study focused on sepsis, we note that these biomarkers, such as SFT2D1, have also been implicated in other disease contexts. For example, recent studies have linked SFT2D1 to tumor progression and immune modulation in cervical and pancreatic cancers (Kang et al., 2024; Liu D. et al., 2023), including associations with alternative splicing events and cellular transport pathways. Although these findings highlight the broader biological relevance of SFT2D1, they also suggest that its expression may be influenced by other conditions. Future work including disease control cohorts will be important to further assess the specificity and diagnostic value of these biomarkers in sepsis.

Finally, although the binding ability between drugs and biomarkers has been predicted by bioinformatics. However, the binding prediction results derived from computer simulations have not been validated by *in vitro* experiments. And due to the lack of validation by *in vitro* experiments, it is not possible to fully determine the binding between biomarkers and drugs in real physiological environments.

## 5 Conclusion

This study identified *BLOC1S1*, *NDUFA1*, and *SFT2D1* as histone acetylation-related biomarkers for sepsis and validated their expression in THP-1 cell models and patient blood samples. Functional analysis demonstrated that inhibition of *SFT2D1* significantly reduced inflammatory cytokine expression, highlighting its potential as a therapeutic target. These findings provide new perspectives for early diagnosis, immune regulation, and targeted therapy in sepsis. Further experimental and clinical research is required to fully elucidate the role of these biomarkers and translate them into clinical applications.

## Data Availability

The datasets analyzed in this study are publicly available from the Gene Expression Omnibus (GEO) under the following accession numbers: GSE95233, GSE65682, and GSE167363. Additional processed data and codes supporting this study are available upon reasonable request from the corresponding authors.

## Supplementary material

SUPPLEMENTARY FIGURE S1 The results of Mendelian randomization (MR) analysis of *SFT2D1* and sepsis. **(A)** the forest plot showed that the effect size of *SFT2D1* on sepsis. **(B)** scatter plot showed the genetic association between *SFT2D1* and sepsis. **(C)** funnel plot showed the no heterogeneity of *SFT2D1* and sepsis. **(D)** leave-one-out analysis for *SFT2D1* on sepsis.

SUPPLEMENTARY FIGURE S2 Data filtering analysis in the GSE167363. **(A)** single-cell RNA sequencing (scRNA-seq) data filtering results in GSE167363. **(B)** filtering of highly variable genes. **(C)** principal component analysis (PCA), the top 30 principal components were selected for subsequent analysis.

## Abbreviations

AUC, area under the curve; ATCC, American type culture collection; DEGs, differentially expressed genes; GEO, gene expression Omnibus; GO, gene ontology; GSEA, gene set enrichment analysis; GSVA, gene set variation analysi; HARGs, histone acetylation-related genes; IVs, instrumental variables; KEGG, Kyoto Encyclopedia of Genes and Genomes; LASSO, least absolute shrinkage and selection operator; LPS, lipopolysaccharide; lncRNAs, long non-coding RNAs; MR, Mendelian randomization; miRNAs, microRNAs; OR, odds ratio; PMA, phorbol 12-myristate 13-acetate; PPI, protein-protein interaction; ROC, receiver operating characteristic; RT-qPCR, reverse transcription-quantitative polymerase chain reaction; SVM-RFE, support vector machine-recursive feature elimination; SiRNA, small interfering RNA; scRNA-seq, single-cell RNA sequencing; SNPs, single nucleotide polymorphisms; TFs, transcription factors; WGCNA, weighted gene co-expression network analysis.
