# Supplementary material for: Unraveling the role of histone acetylation in sepsis biomarker discovery
Source: Front Mol Biosci. 2025 Apr 30;12:1582181. doi: 10.3389/fmolb.2025.1582181 (PMC12074977; doi:10.3389/fmolb.2025.1582181)
Supplement: Supplementary file 1 [file Table1.docx]

**Table S1. The sample information in the GSE95233 dataset**

| **Sample GEO Accession** | **Sample Source Name** | **Age** | **Gender** | **Sample Collection Time** |
| --- | --- | --- | --- | --- |
| GSM2500349 | Control | 52 | F | NR |
| GSM2500350 | Control | 53 | F | NR |
| GSM2500351 | Control | 52 | F | NR |
| GSM2500352 | Control | 53 | F | NR |
| GSM2500353 | Control | 76 | F | NR |
| GSM2500354 | Control | 50 | F | NR |
| GSM2500355 | Control | 57 | F | NR |
| GSM2500356 | Control | 61 | F | NR |
| GSM2500357 | Control | 51 | F | NR |
| GSM2500358 | Control | 52 | F | NR |
| GSM2500359 | Control | 67 | F | NR |
| GSM2500360 | Control | 61 | M | NR |
| GSM2500361 | Control | 57 | M | NR |
| GSM2500362 | Control | 60 | M | NR |
| GSM2500363 | Control | 57 | M | NR |
| GSM2500364 | Control | 67 | M | NR |
| GSM2500365 | Control | 56 | M | NR |
| GSM2500366 | Control | 62 | M | NR |
| GSM2500367 | Control | 52 | M | NR |
| GSM2500368 | Control | 57 | M | NR |
| GSM2500369 | Control | 60 | M | NR |
| GSM2500370 | Control | 59 | M | NR |
| GSM2500371 | Sepsis | 80 | M | Day 1 |
| GSM2500372 | Sepsis | 80 | M | Day 2 |
| GSM2500373 | Sepsis | 59 | M | Day 1 |
| GSM2500374 | Sepsis | 59 | M | Day 3 |
| GSM2500375 | Sepsis | 68 | F | Day 1 |
| GSM2500376 | Sepsis | 68 | F | Day 2 |
| GSM2500377 | Sepsis | 36 | M | Day 1 |
| GSM2500378 | Sepsis | 36 | M | Day 3 |
| GSM2500379 | Sepsis | 66 | M | Day 1 |
| GSM2500380 | Sepsis | 66 | M | Day 3 |
| GSM2500381 | Sepsis | 72 | M | Day 1 |
| GSM2500382 | Sepsis | 72 | M | Day 3 |
| GSM2500383 | Sepsis | 60 | M | Day 1 |
| GSM2500384 | Sepsis | 60 | M | Day 3 |
| GSM2500385 | Sepsis | 81 | M | Day 1 |
| GSM2500386 | Sepsis | 81 | M | Day 3 |
| GSM2500387 | Sepsis | 78 | F | Day 1 |
| GSM2500388 | Sepsis | 78 | F | Day 3 |
| GSM2500389 | Sepsis | 65 | M | Day 1 |
| GSM2500390 | Sepsis | 65 | M | Day 2 |
| GSM2500391 | Sepsis | 37 | M | Day 1 |
| GSM2500392 | Sepsis | 37 | M | Day 3 |
| GSM2500393 | Sepsis | 66 | M | Day 1 |
| GSM2500394 | Sepsis | 66 | M | Day 2 |
| GSM2500395 | Sepsis | 80 | M | Day 1 |
| GSM2500396 | Sepsis | 80 | M | Day 2 |
| GSM2500397 | Sepsis | 83 | M | Day 1 |
| GSM2500398 | Sepsis | 83 | M | Day 3 |
| GSM2500399 | Sepsis | 34 | F | Day 1 |
| GSM2500400 | Sepsis | 34 | F | Day 3 |
| GSM2500401 | Sepsis | 27 | F | Day 1 |
| GSM2500402 | Sepsis | 27 | F | Day 2 |
| GSM2500403 | Sepsis | 55 | M | Day 1 |
| GSM2500404 | Sepsis | 55 | M | Day 2 |
| GSM2500405 | Sepsis | 76 | M | Day 1 |
| GSM2500406 | Sepsis | 76 | M | Day 3 |
| GSM2500407 | Sepsis | 66 | M | Day 1 |
| GSM2500408 | Sepsis | 66 | M | Day 2 |
| GSM2500409 | Sepsis | 42 | F | Day 1 |
| GSM2500410 | Sepsis | 42 | F | Day 2 |
| GSM2500411 | Sepsis | 76 | F | Day 1 |
| GSM2500412 | Sepsis | 76 | F | Day 3 |
| GSM2500413 | Sepsis | 75 | M | Day 1 |
| GSM2500414 | Sepsis | 75 | M | Day 3 |
| GSM2500415 | Sepsis | 54 | M | Day 1 |
| GSM2500416 | Sepsis | 54 | M | Day 3 |
| GSM2500417 | Sepsis | 69 | M | Day 1 |
| GSM2500418 | Sepsis | 69 | M | Day 3 |
| GSM2500419 | Sepsis | 65 | F | Day 1 |
| GSM2500420 | Sepsis | 65 | F | Day 3 |
| GSM2500421 | Sepsis | 63 | M | Day 1 |
| GSM2500422 | Sepsis | 63 | M | Day 3 |
| GSM2500423 | Sepsis | 76 | M | Day 1 |
| GSM2500424 | Sepsis | 76 | M | Day 3 |
| GSM2500425 | Sepsis | 85 | F | Day 1 |
| GSM2500426 | Sepsis | 85 | F | Day 2 |
| GSM2500427 | Sepsis | 25 | M | Day 1 |
| GSM2500428 | Sepsis | 25 | M | Day 3 |
| GSM2500429 | Sepsis | 70 | M | Day 1 |
| GSM2500430 | Sepsis | 70 | M | Day 2 |
| GSM2500431 | Sepsis | 53 | F | Day 1 |
| GSM2500432 | Sepsis | 53 | F | Day 3 |
| GSM2500433 | Sepsis | 70 | M | Day 1 |
| GSM2500434 | Sepsis | 70 | M | Day 2 |
| GSM2500435 | Sepsis | 58 | M | Day 1 |
| GSM2500436 | Sepsis | 58 | M | Day 3 |
| GSM2500437 | Sepsis | 67 | M | Day 1 |
| GSM2500438 | Sepsis | 67 | M | Day 3 |
| GSM2500439 | Sepsis | 85 | M | Day 1 |
| GSM2500440 | Sepsis | 85 | M | Day 2 |
| GSM2500441 | Sepsis | 67 | F | Day 1 |
| GSM2500442 | Sepsis | 67 | F | Day 2 |
| GSM2500443 | Sepsis | 47 | M | Day 1 |
| GSM2500444 | Sepsis | 47 | M | Day 2 |
| GSM2500445 | Sepsis | 65 | M | Day 1 |
| GSM2500446 | Sepsis | 65 | M | Day 2 |
| GSM2500447 | Sepsis | 66 | H | Day 1 |
| GSM2500448 | Sepsis | 66 | H | Day 2 |
| GSM2500449 | Sepsis | 47 | F | Day 1 |
| GSM2500450 | Sepsis | 47 | F | Day 3 |
| GSM2500451 | Sepsis | 48 | F | Day 1 |
| GSM2500452 | Sepsis | 48 | F | Day 2 |
| GSM2500453 | Sepsis | 45 | F | Day 1 |
| GSM2500454 | Sepsis | 45 | F | Day 2 |
| GSM2500455 | Sepsis | 59 | M | Day 1 |
| GSM2500456 | Sepsis | 59 | M | Day 3 |
| GSM2500457 | Sepsis | 78 | M | Day 1 |
| GSM2500458 | Sepsis | 78 | M | Day 3 |
| GSM2500459 | Sepsis | 55 | F | Day 1 |
| GSM2500460 | Sepsis | 55 | F | Day 3 |
| GSM2500461 | Sepsis | 70 | F | Day 1 |
| GSM2500462 | Sepsis | 70 | F | Day 3 |
| GSM2500463 | Sepsis | 72 | H | Day 1 |
| GSM2500464 | Sepsis | 72 | H | Day 3 |
| GSM2500465 | Sepsis | 46 | M | Day 1 |
| GSM2500466 | Sepsis | 46 | M | Day 3 |
| GSM2500467 | Sepsis | 78 | F | Day 1 |
| GSM2500468 | Sepsis | 78 | F | Day 2 |
| GSM2500469 | Sepsis | 53 | F | Day 1 |
| GSM2500470 | Sepsis | 53 | F | Day 3 |
| GSM2500471 | Sepsis | 54 | F | Day 1 |
| GSM2500472 | Sepsis | 54 | F | Day 3 |

Abbreviations: NR, not reported. F, female. M, male
