# Supplementary figures and images for: Unraveling the role of histone acetylation in sepsis biomarker discovery
Source: Front Mol Biosci. 2025 Apr 30;12:1582181. doi: 10.3389/fmolb.2025.1582181 (PMC12074977; doi:10.3389/fmolb.2025.1582181)

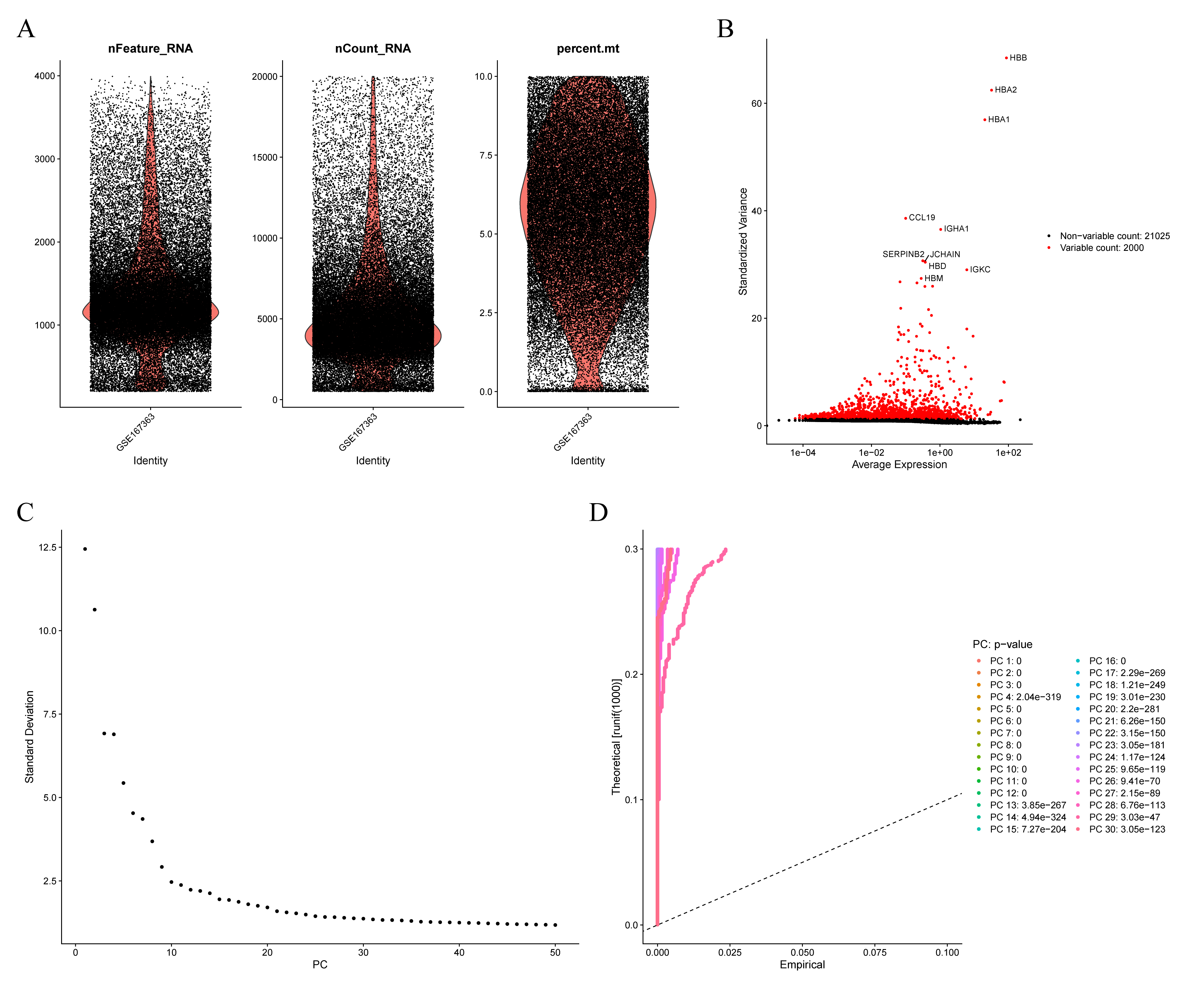

Supplement: Supplementary file 2 [file Image2.tif]

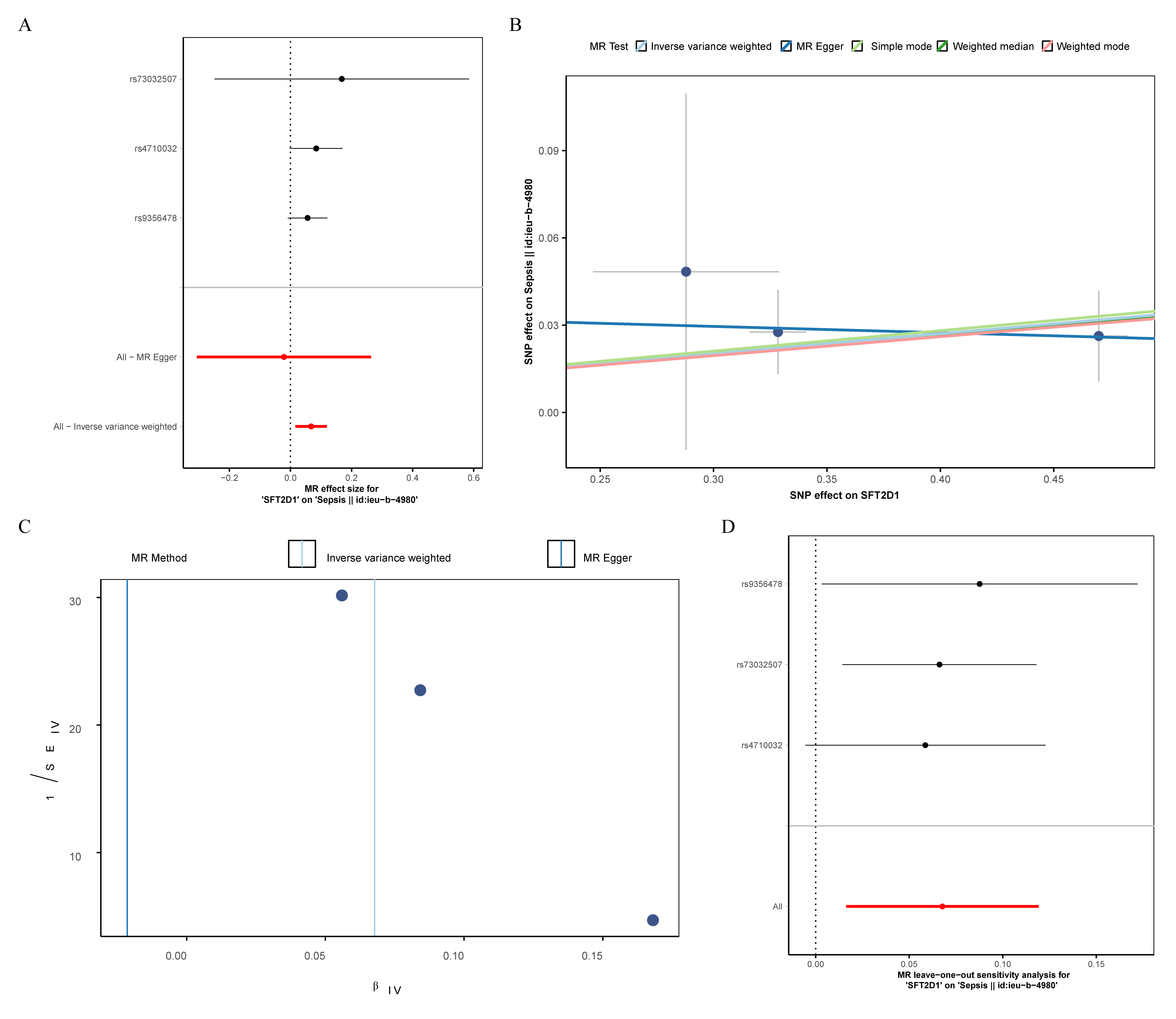

Supplement: Supplementary file 3 [file Image1.tif]
