# Supplementary material for: Unraveling the role of histone acetylation in sepsis biomarker discovery
Source: Front Mol Biosci. 2025 Apr 30;12:1582181. doi: 10.3389/fmolb.2025.1582181 (PMC12074977; doi:10.3389/fmolb.2025.1582181)
Supplement: Supplementary file 4 [file Table2.docx]

**Table S2. The sample information in the GSE65682 dataset**

| **Sample GEO Accession** | **Sample Source Name** | **Age** | **Gender** |
| --- | --- | --- | --- |
| GSM1691902 | Control | 63 | male |
| GSM1691913 | Control | 24 | female |
| GSM1691917 | Control | 75 | male |
| GSM1691920 | Control | 73 | male |
| GSM1691926 | Control | 66 | female |
| GSM1691936 | Control | 49 | male |
| GSM1691952 | Control | 53 | male |
| GSM1692053 | Control | 75 | male |
| GSM1692054 | Control | 77 | male |
| GSM1692075 | Control | 82 | female |
| GSM1692078 | Control | 70 | male |
| GSM1692080 | Control | 30 | male |
| GSM1692110 | Control | 41 | female |
| GSM1692147 | Control | 65 | male |
| GSM1692150 | Control | 64 | female |
| GSM1692155 | Control | 77 | male |
| GSM1692176 | Control | 42 | female |
| GSM1692195 | Control | 74 | male |
| GSM1692219 | Control | 74 | male |
| GSM1692231 | Control | 59 | female |
| GSM1692239 | Control | 64 | male |
| GSM1692248 | Control | 73 | male |
| GSM1692249 | Control | 62 | male |
| GSM1692270 | Control | 55 | female |
| GSM1692275 | Control | 86 | male |
| GSM1692281 | Control | 68 | male |
| GSM1692314 | Control | 54 | male |
| GSM1692321 | Control | 68 | male |
| GSM1692328 | Control | 73 | male |
| GSM1692411 | Control | 67 | male |
| GSM1692419 | Control | 48 | male |
| GSM1692442 | Control | 46 | female |
| GSM1692457 | Control | 59 | female |
| GSM1692463 | Control | 56 | female |
| GSM1692468 | Control | 54 | male |
| GSM1692474 | Control | 70 | male |
| GSM1692475 | Control | 54 | female |
| GSM1692476 | Control | 65 | female |
| GSM1692483 | Control | 56 | female |
| GSM1692490 | Control | 66 | male |
| GSM1692496 | Control | 53 | male |
| GSM1692502 | Control | 81 | female |
| GSM1691871 | Sepsis | 50 | female |
| GSM1691877 | Sepsis | 61 | male |
| GSM1691901 | Sepsis | 31 | female |
| GSM1691954 | Sepsis | 63 | male |
| GSM1691959 | Sepsis | 63 | male |
| GSM1691968 | Sepsis | 53 | female |
| GSM1691980 | Sepsis | 77 | female |
| GSM1691986 | Sepsis | 51 | female |
| GSM1692007 | Sepsis | 74 | female |
| GSM1692016 | Sepsis | 48 | male |
| GSM1692022 | Sepsis | 64 | male |
| GSM1692030 | Sepsis | 62 | male |
| GSM1692038 | Sepsis | 56 | female |
| GSM1692050 | Sepsis | 38 | female |
| GSM1692059 | Sepsis | 71 | female |
| GSM1692066 | Sepsis | 49 | female |
| GSM1692084 | Sepsis | 71 | male |
| GSM1692086 | Sepsis | 67 | male |
| GSM1692114 | Sepsis | 68 | male |
| GSM1692117 | Sepsis | 64 | male |
| GSM1692119 | Sepsis | 82 | female |
| GSM1692126 | Sepsis | 82 | male |
| GSM1692137 | Sepsis | 64 | female |
| GSM1692138 | Sepsis | 67 | male |
| GSM1692160 | Sepsis | 48 | male |
| GSM1692166 | Sepsis | 40 | male |
| GSM1692198 | Sepsis | 54 | male |
| GSM1692201 | Sepsis | 66 | female |
| GSM1692211 | Sepsis | 75 | female |
| GSM1692238 | Sepsis | 75 | male |
| GSM1692255 | Sepsis | 64 | male |
| GSM1692261 | Sepsis | 60 | male |
| GSM1692278 | Sepsis | 62 | male |
| GSM1692302 | Sepsis | 49 | female |
| GSM1692329 | Sepsis | 82 | female |
| GSM1692330 | Sepsis | 57 | male |
| GSM1692337 | Sepsis | 66 | female |
| GSM1692362 | Sepsis | 65 | female |
| GSM1692364 | Sepsis | 42 | female |
| GSM1692372 | Sepsis | 65 | male |
| GSM1692379 | Sepsis | 73 | female |
| GSM1692384 | Sepsis | 79 | male |
| GSM1692388 | Sepsis | 68 | male |
| GSM1692397 | Sepsis | 73 | male |
| GSM1692412 | Sepsis | 71 | female |
| GSM1692416 | Sepsis | 40 | female |
| GSM1692424 | Sepsis | 68 | male |
| GSM1692453 | Sepsis | 60 | male |
| GSM1692459 | Sepsis | 56 | female |
| GSM1692477 | Sepsis | 54 | male |
| GSM1692501 | Sepsis | 67 | female |
