# Supplementary material for: Unraveling the role of histone acetylation in sepsis biomarker discovery
Source: Front Mol Biosci. 2025 Apr 30;12:1582181. doi: 10.3389/fmolb.2025.1582181 (PMC12074977; doi:10.3389/fmolb.2025.1582181)
Supplement: Supplementary file 6 [file Table3.docx]

**Table S3. The sample information in the GSE167363 dataset**

| **Sample GEO Accession** | **Sample Source Name** | **Sample Collection Time** |
| --- | --- | --- |
| [GSM5102900](https://www.ncbi.nlm.nih.gov/geo/query/acc.cgi?acc=GSM5102900) | control | NR |
| [GSM5102901](https://www.ncbi.nlm.nih.gov/geo/query/acc.cgi?acc=GSM5102901) | control | NR |
| [GSM5102902](https://www.ncbi.nlm.nih.gov/geo/query/acc.cgi?acc=GSM5102902) | Sepsis | 0 hours |
| [GSM5102903](https://www.ncbi.nlm.nih.gov/geo/query/acc.cgi?acc=GSM5102903) | Sepsis | 6 hours |
| [GSM5102904](https://www.ncbi.nlm.nih.gov/geo/query/acc.cgi?acc=GSM5102904) | Sepsis | 0 hours |
| [GSM5102905](https://www.ncbi.nlm.nih.gov/geo/query/acc.cgi?acc=GSM5102905) | Sepsis | 6 hours |
| [GSM5511351](https://www.ncbi.nlm.nih.gov/geo/query/acc.cgi?acc=GSM5511351) | Sepsis | 0 hours |
| [GSM5511352](https://www.ncbi.nlm.nih.gov/geo/query/acc.cgi?acc=GSM5511352) | Sepsis | 6 hours |
| [GSM5511353](https://www.ncbi.nlm.nih.gov/geo/query/acc.cgi?acc=GSM5511353) | Sepsis | 0 hours |
| [GSM5511354](https://www.ncbi.nlm.nih.gov/geo/query/acc.cgi?acc=GSM5511354) | Sepsis | 6 hours |
| [GSM5511355](https://www.ncbi.nlm.nih.gov/geo/query/acc.cgi?acc=GSM5511355) | Sepsis | 0 hours |
| [GSM5511356](https://www.ncbi.nlm.nih.gov/geo/query/acc.cgi?acc=GSM5511356) | Sepsis | 6 hours |
